# Water Extract of *Cryphaea heteromalla* (Hedw.) D. Mohr Bryophyte as a Natural Powerful Source of Biologically Active Compounds

**DOI:** 10.3390/ijms20225560

**Published:** 2019-11-07

**Authors:** Fiorenza Provenzano, Jesús Lozano Sánchez, Estella Rao, Radha Santonocito, Lorena Anna Ditta, Isabel Borrás Linares, Rosa Passantino, Patrizia Campisi, Maria Giovanna Dia, Maria Assunta Costa, Antonio Segura-Carretero, Pier Luigi San Biagio, Daniela Giacomazza

**Affiliations:** 1 Istituto di Biofisica, Consiglio Nazionale delle Ricerche, Via U. La Malfa, 153, 90146 Palermo, Italy; fiorenza.provenzano@pa.ibf.cnr.it (F.P.); estella.rao@pa.ibf.cnr.it (E.R.); radha.santonocito@pa.ibf.cnr.it (R.S.); lorenaanna.ditta@pa.ibf.cnr.it (L.A.D.); rosa.passantino@cnr.it (R.P.); daniela.giacomazza@cnr.it (D.G.); 2 Department of Food Science and Nutrition, University of Granada, Campus Universitario s/n, 18071 Granada, Spain; jesusls@ugr.es; 3 Center of Research and Development of Functional Food, Health Science Technological Park, Avda. del Conocimiento s/n, 18100 Granada, Spain; iborras@ugr.es (I.B.L.); ansegura@ugr.es (A.S.-C.); 4 Dipartimento di Scienze e Tecnologie Biologiche, Chimiche e Farmaceutiche, Università di Palermo, Via Archirafi 38, 90123 Palermo, Italy; patrizia.campisi@unipa.it (P.C.);; 5 Department of Analytical Chemistry, Faculty of Sciences, University of Granada, 18071 Granada, Spain

**Keywords:** bryophytes, *Cryphaea heteromalla*, extract HPLC-ESI-TOF-MS analysis, oxidative stress evaluation

## Abstract

Bryophytes comprise of the mosses, liverworts, and hornworts. *Cryphaea heteromalla,* (Hedw.) D. Mohr, is a non-vascular lower plant belonging to mosses group. To the date, the most chemically characterized species belong to the liverworts, while only 3.2% and 8.8% of the species belonging to the mosses and hornworts, respectively, have been investigated. In this work, we present Folin–Ciocalteu and oxygen radical absorbance capacity (ORAC) data related to crude extracts of *C. heteromalla* obtained by three different extraction solvents: pure water (WT), methanol:water (80:20 *v*/*v*) (MET), and ethanol:water (80:20 *v*/*v*) (ETH). The water extract proved to be the best solvent showing the highest content of biophenols and the highest ORAC value. The *C. heteromalla*-WT extract was investigated by HPLC-TOF/MS (High Performance Liquid Chromatography-Time of Flight/Mass Spectrometry) allowing for the detection of 14 compounds, five of which were phenolic compounds, derivatives of benzoic, caffeic, and coumaric acids. Moreover, the *C. heteromalla* WT extract showed a protective effect against reactive oxygen species (ROS) generation induced by tert-butyl hydroperoxide (TBH) on the murine NIH-3T3 fibroblast cell line.

## 1. Introduction

Bryophytes, long time neglected for their reduced dimensions, are generally considered as the first plants to appear on land [1], thus, they can be considered the closest relatives of early terrestrial plants. They are taxonomically placed between algae and pteridophytes. More than 20,000 bryophyte species are present worldwide [2] and further distributed into three groups: mosses (*Musci*), liverworts (*Hepaticae*), and the hornworts (*Anthocerotae*). Since ancient times, bryophytes have been used for various applications. Native Americans used them for drugs, fibers, and clothing [3], while in the Chinese practice, they have been one of the main components to treat cardiovascular and pulmonary diseases and skin infections [4].

Bryophytes populate different kinds of habitats, both, humid and desert environments [5], with the exception of seas and oceans [3]. They also represent the main flora in unfavorable areas, such as the tundra or Antarctica [2], and are particularly important for the maintenance of these ecosystems [3]. Their attachment to soil or other substrates is attained by hair-like structures called rhizoids. Bryophytes are devoid of a complete system for the transport of water and nutrients, normally introduced by leaves, and for this reason they generally possess a small size, although some species can reach a few meters [5].

Possessing a thin cuticle, bryophytes are particularly sensitive to external agents and for this reason they have been used to monitor atmospheric pollutants, mainly sulfur dioxide and nitrogen oxide [6]. An important characteristic of these plants is dehydration resistance, although this is extremely variable between different species. In the absence of water, they can lose almost all their total water content without any damage to cell and organelle integrity [3]. Bryophytes have a good tolerance to the accumulation of heavy metals, such as lead or zinc, thus, they can be employed as good indicators of environmental pollution [7,8,9]. Furthermore, bryophytes contain strong antioxidant properties that allow them to deal with extreme stress conditions [10]. Lastly, some bryophyte species have also shown antimicrobial activity against some common bacteria responsible for epidermal infections [11]. All these peculiar features indicate that bryophytes possess a highly-developed defense system against unfavorable environmental conditions.

**Figure 1:**
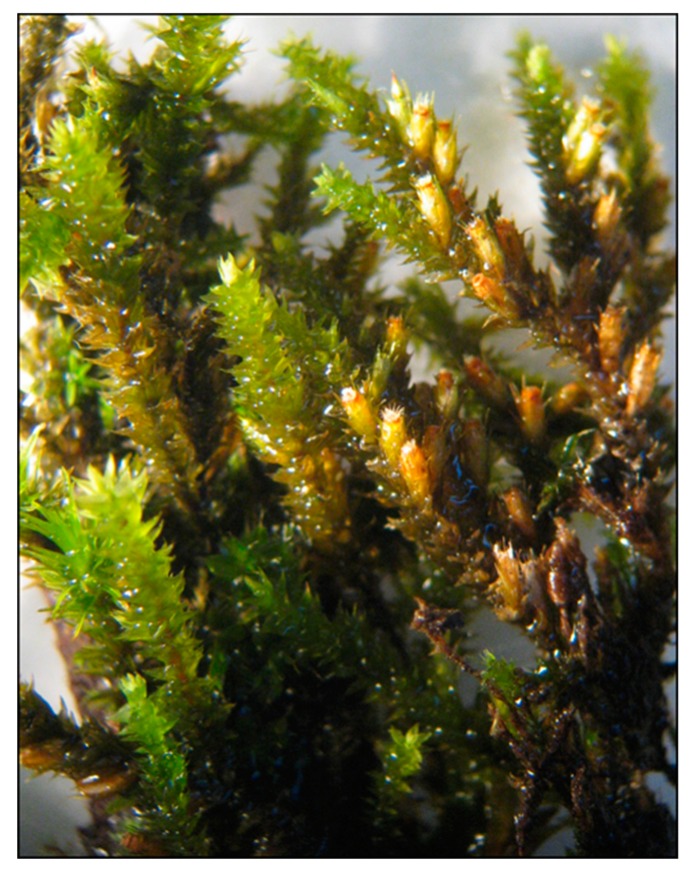
Hydrated plants of *Cryphaea heteromalla* (Hedw.) D. Mohr harvested in western Sicily, Italy, in the Trapani area.

*Cryphaea heteromalla* (Figure 1) belongs to the mosses group and is a pleurocarpous epiphyte growing on the bark of the trees. It is a mountainous species distributed in Europe, Macaronesia, Southwest Asia, and North Africa, and is extensively widespread in Italy [12]. *Cryphaea heteromalla* has primary stems appressed to the substrate and secondary stems 1–3 cm long, projecting stiffly away from the primary stem. The leaves are 1–1.3 mm long and are imbricate when dry, erect-patent when moist; they are ovate with an acute to acuminate apex and the nerve is stout, extending well over half the length of the lamina. The sporophytes are often numerous, situated on only one side of the secondary stems. The capsules are 2.5–3 mm long, partially covered by the leaves, and the seta are very short, around 0.2 mm long. The plants grow in dark green tufts forming extensive patches largely covering the surface of the trunk. 

It is known that free radicals, such as reactive oxygen species (ROS) and reactive nitrogen species (RNS), are formed during metabolic reactions in living organisms [13]. When free radicals are in excess or unbalanced by the antioxidant cell defense system, they can initiate toxic oxidative reactions leading to the oxidation of proteins, lipids, and nucleic acids, and the alteration of membrane properties, such as fluidity, ion transport, enzyme activities, and protein cross-linking [14]. Therefore, the increasing request for antioxidant molecules has led to the exploration of promising new sources of valuable compounds, and even waste-products [15], for the purpose of enhancing the healthy content of food [16,17] or identifying novel pharmaceutical compounds. The therapeutic administration of antioxidants has been considered, in the last decades, to counteract the unhealthy effects of oxidative stress, and powerful new sources of antioxidant molecules are under investigation. Antioxidants are chemical compounds characterized by the presence of one or more phenolic rings and they able to prevent, slow, or terminate radical formation reactions. 

In the present work, we evaluated, using the Folin–Ciocalteu method, the total biophenol amount extracted in pure water (WT), methanol:water (80:20 *v*/*v*) (MET), and ethanol:water (80:20 *v*/*v*) (ETH), from *C. heteromalla* species harvested in Monte Bonifato, a Sicilian mountain in the Trapani area of Italy. The antioxidant ability of each extract was analyzed by the oxygen radical absorbance capacity (ORAC) assay. As the WT extract showed the highest total biophenol content and the highest ORAC value, the WT extract components were characterized by HPLC-ESI-TOF-MS (High Performance Liquid Chromatography-Time of Flight/Mass Spectrometry) measurements. The *C. heteromalla*-WT extract was added to a NIH-3T3 murine fibroblast cell culture to verify its ability to counteract the damage caused by excessive ROS generation.

## 2. Results

### 2.1. Total Biophenols and the Antioxidant Ability of WT, MET, and ETH Cryphaea heteromalla Extracts

In the last few decades, bryophytes have attracted great interest, and chemicals and secondary metabolites have been identified from several species, but up to now, the phytochemical content and antioxidant activity of *C. heteromalla* species are still unknown. In this work, *C. heteromalla* powder was dissolved in three different extraction solvents: pure water (WT), methanol:water (80:20 *v*/*v*) (MET), and ethanol:water (80:20 *v*/*v*) (ETH). The biophenol amount was evaluated by the Folin–Ciocalteu assay at different extraction times.

In Table 1, the biophenol amounts of the bryophyte extracts in WT, MET, and ETH, after incubation up to 4 days, are reported. The results indicate that the WT extract contained the highest biophenol concentration showing that water was the most suitable solvent for biophenol extraction, in agreement with the literature data obtained from different bryophyte mosses and plants [18,19,20]. Moreover, no further biophenol content increase was observed after three days and, on this basis, further experiments were performed after 3-day incubation.

**Figure 2:**
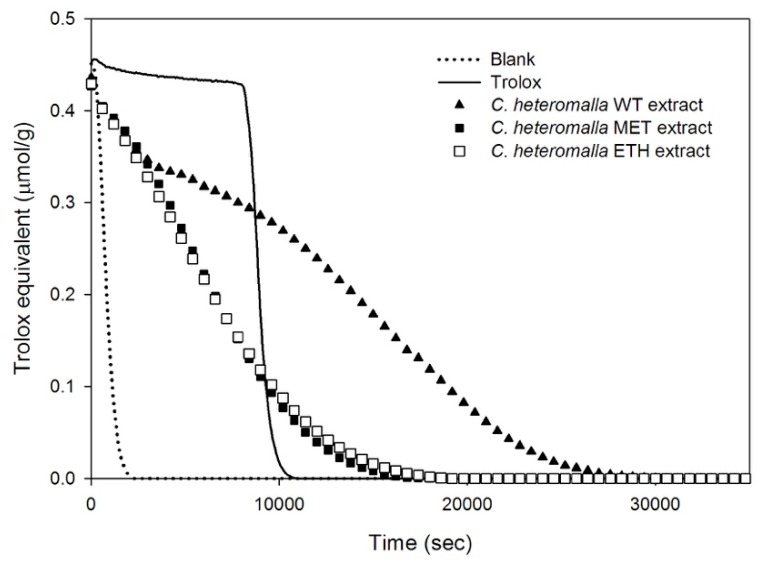
Antioxidant power measured by the oxygen radical absorbance capacity (ORAC) curves. ORAC curves as a function of time for the evaluation of the antioxidant power of *Cryphaea heteromalla* extracts after 3-day incubation in MET (full squares), ETH (empty squares), and WT (full triangles). Trolox, used as a reference, and blank solution are reported as continuous and dotted lines, respectively. The three blank solutions, in WT, MET, and ETH were perfectly coincident, thus, in the figure only, the WT blank is reported.

The well-known ability of the biophenols to scavenge free radicals and ROS [21,22,23] led us to investigate the antioxidant activity of *C. heteromalla* bryophyte extracts. To evaluate the capacity of our extracts to counteract free radicals, the ORAC assay was performed. In this test, the fluorescence decay of the fluorescein, induced by the presence of the free radical initiator (AAPH), is counteracted by the antioxidant activity of the samples or the Trolox, the latter is used as a reference compound. Thus, the higher the antioxidant power of the sample, the larger the area underneath its fluorescence signal. In Figure 2, the fluorescence decay of the samples obtained after 3-day extraction time in pure water, methanol, and ethanol solutions are reported. The sample in water exhibited the larger fluorescence decay curve, while the decay attained in the case of the samples in MET and ETH solutions was lower and comparable for both samples (Figure 2). Data were in agreement with the results achieved by the Folin–Ciocalteu assay.

In Table 2, the ORAC values obtained as a function of the extraction time in WT, MET, and ETH are reported. Once more, it was confirmed that a prolonged incubation in water is able to extract a higher concentration of active molecules. 

### 2.2. Chemical Characterization of the Cryphaea heteromalla WT Extract

**Figure 3:**
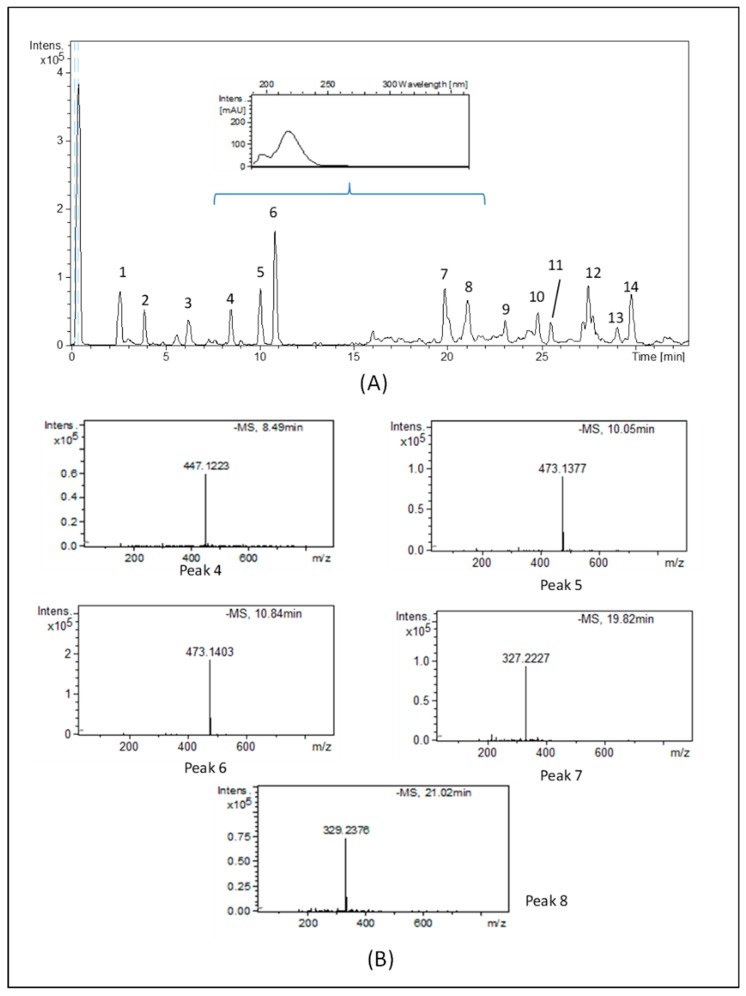
HPLC-ESI-TOF-MS analysis. (**A**) Base peak chromatogram (BPC) of the *Cryphaea heteromalla* WT extract obtained by the HPLC-ESI-TOF-MS. (**B**) MS spectra of the main phenolic compounds identified in the *Cryphaea heteromalla* extract.

As our results showed that the WT extract contained the higher biophenol amount and exhibited the highest ORAC value, the 3-day incubation WT extract was chosen for further investigations. The resulting base peak chromatogram (BPC) for the *C. heteromalla* WT extract obtained by the HPLC-ESI-TOF-MS method is shown in Figure 3A. 

The proposed elemental composition for all compounds with their retention times (RT), the experimental and calculated *m*/*z*, the molecular formula, error (ppm), and milliSigma value (mSigma) are compiled in Table 3. These compounds have been numbered according to their elution order.

Molecular formulae were based upon an interpretation of their MS spectra provided by TOF-MS. The analysis of the true isotopic pattern by ESI-TOF-MS in combination with excellent mass resolution and mass accuracy is the perfect choice for molecular formula determination using the Generate Molecular Formula Editor. To identify the chemical compounds, a low tolerance of 0.2 and a low error (≤20 ppm) were chosen. 

The HPLC-ESI-TOF-MS technique allowed for the detection of 14 compounds. The ions detected were tentatively identified according to their generated molecular formula after a thorough survey of the literature. A total of five phenolic compounds were tentatively identified in the *C. heteromalla* bryophyte extract. Indeed, benzoic, caffeic, and *p*-coumaric acid derivatives were detected in the samples. Concerning the benzoic acid derivatives, compound no. 4 was tentatively assigned as di-hydroxybenzoic acid hexoside-pentoside. This compound has previously been described in plants [24]. With regard to caffeic acid derivatives, two isomers were proposed. The first isomer (compound no. 5, eluting at 10.03 min) produced the base MS peak at *m*/*z* 473, characteristic of caffeic acid hexoside-pentoside or isomer. Molecular ion [M–H]- at *m*/*z* 473 and the same molecular formula (C_20_H_25_O_13_) was found for peak 6, eluting at 10.8 min and corresponding to the second caffeic acid derivative isomer. Several authors have characterized these compounds in plants [25,26,27,28]. Concerning *p*-coumaric acid derivatives, two chromatographic peaks corresponded to molecules of *p*-coumaric acid linked to hesoxide. Peaks no. 7 and no. 8, with *m*/*z* 327 and 329, and molecular formula C_18_H_31_O_5_ and C_18_H_33_O_5_, were tentatively characterized as dihydro-*p*-coumaric acid hexoside and its hydrated derivative, respectively [25,26,27]. In the inset of Figure 3A, the UV spectra of the compounds described above are reported. Molecules showed the absorbance maxima at 230 nm, which is the typical wavelength of phenolic groups. The MS spectra of the main phenolic compounds identified in the sample are summarized in Figure 3B. 

Besides biophenols, other compounds were also detected in the samples which have been associated with a variety of biological functions in plants. Bryophyte plants produce a significant level of highly unsaturated fatty acids [2]. Oxylipins are bioactive metabolites derived from the oxygenation of polyunsaturated fatty acids. They have not received much attention compared to other plant metabolites, probably because they are not abundant, making their difficult detection. Nevertheless, oxylipin metabolites belonging to the subclass of jasmonates have been recently found in plants [29]. Analysis of the samples allowed for the detection of five compounds within this class. Compound numbers 9, 10, and 13 were identified as cyclopentenone oxylipin derivatives. Compound 9 and 10 yielded deprotonated molecules at *m*/*z* 239 and 237, which are tentatively identified as hydrated-OPC-4 (hydrated-3-oxo-2-(pent-2-enyl)-cyclopentane-1-butanoic acid) and OPC-4 (3-oxo-2-(pent-2-enyl)-cyclopentane-1-butanoic acid). Peak no. 13 showed a molecular ion at *m*/*z* 265. Its MS spectra generated the molecular formula C_16_H_25_O_3_, which is described as OPC-6 (3-oxo-2-(2-(Z)-pentenyl)cyclopentane-1-hexanoic acid). These compounds (peak numbers 9, 10, and 13) have previously been described in plants [30,31,32]. The spectra generated for peak 11 and 14 gave deprotonated molecules at *m*/*z* 263 and 291, respectively, which could be attributed to derivatives of phytodienoic acid. Compound 11 was proposed as its dinor-oxo-phytodienoic acid form. It eluted before 12-oxo-phytodienoic acid (no. 14), which is a biologically active, immediate precursor of jasmonic acids [33,34]. Jasmonates are able to regulate the plant defense against wounds and pathogen attack, promote reproductive development, and inhibit vegetative growth [35]. In the last decade, their anticancer activity has been associated with several murine and human cancer types [36]. 

### 2.3. WT Extract Protective Effect against Oxidative Stress

**Figure 4:**
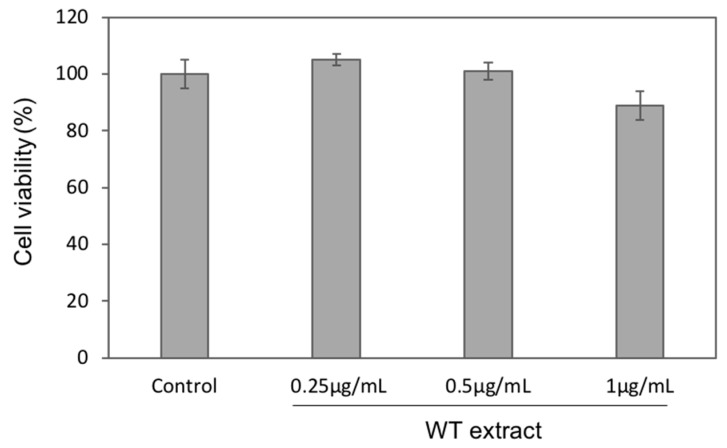
WT extract effects on NIH-3T3 cell viability. NIH-3T3 cells were treated with 0.25, 0.5, or 1.0 µg/mL of *C. heteromalla* WT extract for 24 h. Cell viability of each sample is expressed as the percentage between treated and untreated (Control) cells. The data are shown as the means ± SD of at least three separate experiments.

The murine fibroblast NIH-3T3 cell line was used as a cellular model to mimic oxidative stress and to investigate the WT extract antioxidant ability. Before analyzing the WT extract antioxidant effect, three different concentrations (0.25, 0.5, and 1 µg/mL) of the extract were tested to assess any cytotoxic effect by the MTS viability assay. As reported in Figure 4, a very slight cell viability decrease was observed for the highest concentration (1 µg/mL), whereas for the other two concentrations (0.25 and 0.5 µg/mL), cell viability was comparable to the untreated cells (Control).

**Figure 5:**
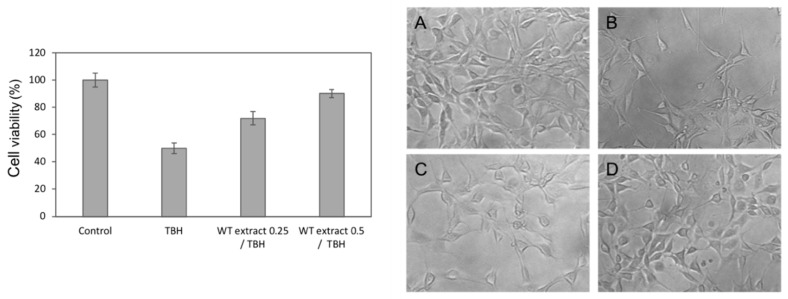
The *Cryphaea heteromalla* WT extract protective effect against tert-butyl hydroperoxide (TBH)-induced reactive oxygen species (ROS). Left panel. NIH-3T3 cells were untreated (Control) or treated overnight with only TBH at 250 µM concentration (TBH) or with the *C. heteromalla* WT extract at the concentration of 0.25 or 0.5 µg/mL along with the 250 µM TBH (WT extract 0.25/TBH and WT extract 0.5/TBH, respectively). The data are shown as the means ± SD of at least three separate experiments. Right panel. Microscope images of the morphological aspect of the cells after treatment. (**A**) Untreated cells (Control); (**B**) TBH-treated cells; (**C**) cells treated with TBH and 0.25 µM WT extract; (**D**) cells treated with TBH and 0.5 µg/mL WT extract. Original magnification 20×.

Therefore, the concentrations 0.25 and 0.5 µg/mL were used to investigate the ability of WT extract to counteract the oxidative stress induced in NIH-3T3 cells by tert-butyl hydroperoxide (TBH), a direct-acting oxidative stress-inducing agent. As shown by the histogram on the left of Figure 5, cell viability considerably decreased after overnight treatment of the NIH-3T3 cells with 250 µM TBH compared to untreated cells (Control). Conversely, when cells were treated with WT extract and TBH at the same time, an evident cytoprotection exerted by the extract was detected at both the tested concentrations. The cytoprotective effect was particularly pronounced in the case of the highest concentration (0.5 µg/mL) for which cell viability reached 90% (Figure 5, on the left). The WT extract protective effect was also confirmed by microscope observation (Figure 5C,D on the right). 

**Figure 6:**
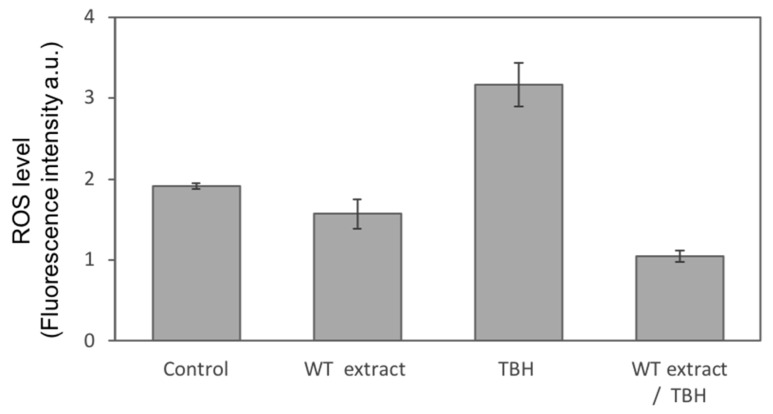
Fluorescent evaluation of the WT extract protective effect against ROS generated by TBH. NIH-3T3 cells were untreated (Control), treated with the *Cryphaea heteromalla* WT extract at the concentration of 0.5 µg/mL (WT extract), treated with 500 µM TBH alone (TBH), or along with 0.5 µg/mL of WT extract (WT extract/TBH). The data are shown as the mean ± SD of at least three separate experiments.

To confirm the protective effect of the *C. heteromalla* WT extract against ROS generation induced by TBH, a DCFH-DA fluorescent analysis was performed. DCFH-DA is a cell-permeable fluorogenic probe that, after crossing the cell membranes, is hydrolyzed by intracellular esterases to non-fluorescent DCFH. In the presence of ROS, DCFH is oxidized to fluorescent DCF giving rise to an intracellular, more or less intense, green fluorescence, depending on the ROS concentration. Our results showed that the overnight pretreatment of NIH-3T3 cells with WT extract, at the nontoxic concentration of 0.5 µg/mL, significantly inhibited more than 50% of the ROS induced by 1-hour treatment with 500 µM TBH. Furthermore, the treatment with the WT extract alone was able to further lower the basal ROS level of the untreated cells (Control) (Figure 6). 

**Figure 7:**
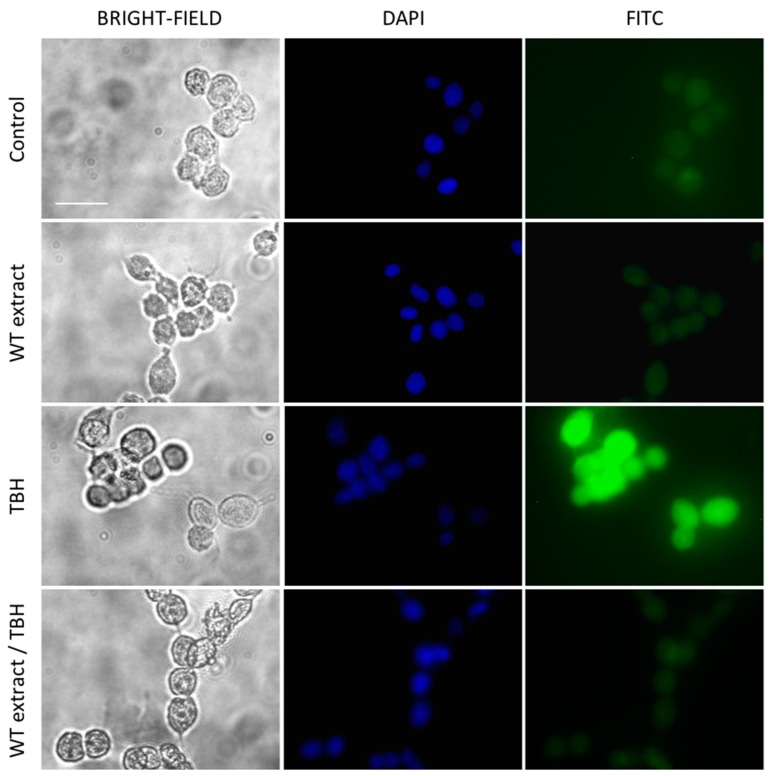
Intracellular ROS detection by fluorescent microscopy. Images of live NIH-3T3 cells untreated (Control), treated overnight with WT extract 0.5 µg/mL (WT extract), treated for 1 h with TBH at the 500 µM concentration (TBH), pretreated with WT extract and then treated with TBH (WT extract/TBH). Original magnification 20×. Scale bar 50 μm.

The intracellular ROS generated by the oxidative stress induced by TBH were also investigated by fluorescence microscopy using the DCFH-DA probe. Results confirmed the high protective effect of WT extract on oxidative stress at the concentration of 0.5 µg/mL. In fact, while cells treated with only TBH showed high green fluorescence, cells pretreated with WT extract before TBH addition presented a very weak fluorescence, comparable to the untreated cells (Control) and cells treated only with WT extract (Figure 7). 

## 3. Discussions

Since ancient times, plants have attracted the attention of people for their applications in several fields of research, especially for medical purposes. Among the huge amount of biomolecules present in plants, biophenols deserve a special place due to their effects against oxidative stress and its unpleasant consequences. Indeed, oxidative stress is involved in the onset of many human pathological conditions, among which neurodegenerative disorders (Alzheimer’s disease (AD) and Parkinson’s disease) [37,38], vascular pathologies [39,40], cancer [41,42], and aging [43]. Thus, the research for natural antioxidants intensified after the discovery that commercial synthetic molecules exhibited toxic effects leading to cancer [44]. HPLC-ESI-TOF-MS analysis indicated that *C. heteromalla* extract contained three derivatives of powerful antioxidant molecules: benzoic, caffeic, and *p*-coumaric acids.

In particular, Modi et al. [45] have demonstrated that in mouse BV-2 microglial cells, sodium benzoate strongly inhibited intracellular ROS formation, increased after addition of LPS, Aβ(1-42) (the 42-residue peptide involved in Alzheimer’s Disease (AD) onset), and 1-methyl-4-phenylpyridinium (a Parkinsonian toxin) through suppression of p21^rac^ signaling. Furthermore, oral administration of sodium benzoate protected neurons, reduced extracellular plaque formation, and improved spatial learning and memory in the AD mouse model [45]. Moreover, it was confirmed that monophenol antioxidant efficiency is strongly enhanced if an additional hydroxyl group is present in the molecules [46]. 

Hydroxycinnamic acid and its derivatives, such as caffeic, *p*-coumaric, and ferulic, play a crucial role in nature. In fact, these secondary metabolites can be linked with amines, amino acids, peptides, esters, sugars derivatives, and glycosides [47]. Caffeic acid possesses anticancer, anti-inflammatory, immune-modulatory, and antioxidant properties [48,49]. The antioxidant, in vitro and in vivo results indicated that caffeic acid can inhibit the activity of lipoxygenase and avoid lipid peroxidation [50,51]. Furthermore, Li et al. [51] established that the administration of caffeic acid in L-02 cell cultures, increased the cell viability after H_2_O_2_ exposure and protected DNA against oxidative damage via extracellular signal-regulated kinase (ERK) signaling. More recently, Yang et al. [52] found that, after tert-butyl hydroperoxide stimulus, the presence of caffeic acid in HepG2 cells promoted the expression of detoxifying enzymes, such as heme oxygenase-1 and glutamate-cysteine ligase, by ERK phosphorylation and Nrf2 activation. Their conclusions indicated that caffeic acid was a suitable chemoprotective agent against oxidative damage in the liver.

The antioxidant properties of caffeic and *p*-coumaric acids were also demonstrated to be effective against some tumor cells, such as colon adenocarcinoma HT29 cells and human melanoma HCT15 cells [53,54] and a concentration-dependent effect in the biophenol range 50–200 μM was observed. A moderate increase of ROS can induce cell proliferation and differentiation; thus, the reduction of free radical generation is important for the maintenance of healthy cells. Furthermore, it is known that cell adhesion is a critical step in the development of tumor metastasis and the lack of proper contacts with the extracellular matrix (ECM), mediated by integrin receptors, could cause cell death. Bouzaiene et al. [53] demonstrated that phenolic acids could affect the attachment of A549 and HT29-D4 tumor cells to the ECM by operating on integrin receptors, with a not well-defined mechanism. 

The total antioxidant activity obtained by the FRAP (ferric reducing antioxidant power) assay suggested that phenolic acids, e.g., caffeic and *p*-coumaric, possessed a high protective effect on the action of human endogenous antioxidant enzymes, such as SOD, catalase, and peroxidase [20]. At the beginning of the current decade, it was observed that hydroxycinnamic derivatives had inhibitory activity against the action of tyrosinase enzymes, catalyzing the oxidation of tyrosine to dopaquinone [55]. This reaction induces, in food, an unpleasant modification in flavor, nutritional properties, and the color of the products [55]. In addition, hydroxycinnamic derivatives, acting also as tyrosinase inhibitors, have found important applications in the cosmetic industry as whitening functional cosmetics in relation to skin hyperpigmentation [56]. Caffeic acid is also able to act in vivo against lipid oxidation and hypercholesterolemia due to oxidative stress induced by iron overload in rats. The administration of caffeic acid sensibly reduced lipid oxidation in the liver cholesterol level while increasing hematic concentration of vitamin E [57]. 

Oral administration of 317 mg/day of *p*-coumaric acid in rats for 30 days caused the lowering of lipid peroxidation and low-density lipoprotein (LDL) cholesterol levels, without affecting HDL cholesterol concentration, demonstrating its ability in the prevention of lipid peroxidation and atherosclerosis [58]. *p*-Coumaric acid was found to be suitable in decreasing the expression of inflammatory mediators, TNF-α and IL-6 cytokines, in arthritic rats. Its effect was also effective in attenuating the symptoms of the disease [59]. Peng et al. indicated that *p*-coumaric acid could be efficient in the prevention of caractogenesis by protecting human lens epithelial cells (SRA01/04) from oxidative stress and apoptosis by acting on the MAPK signaling pathway [60].

In addition to phenolic acids, the *C. heteromalla* extract contained some chemical species of jasmonates. These molecules, that only recently have attracted the attention of the researchers, belong to the protective system of the plants. Although very few data exist concerning the effects of jasmonates on animal/human cells, it has been demonstrated that they had antitumoral activity [36] without being toxic against normal cells [61]. Furthermore, their antioxidant effect was tested in human neuroblastoma SH-SY5Y culture cells [62]. Results indicated that jasmonates, and in particular 12-oxo-phytodienoic acid, suppressed oxidative stress-induced death of human neuroblastoma cells, activating the Nrf2 pathway [62]. Very recently, it was established that at very low concentrations, the order of a few μm/L, jasmonates can reduce the excess of ROS in microglial cells and enhance their phagocytic action. These effects, in turn, can reduce the neuroinflammation process and increase the elimination of dangerous supramolecular structures, such as amyloid aggregates, accumulating in neurodegenerative pathologies [63]. 

All these results suggest that *C. heteromalla* aqueous extract could be a suitable source of bioactive molecules having beneficial effects on human health. 

## 4. Materials and Methods

### 4.1. Chemicals and Reagents

Folin–Ciocalteau solution, sodium carbonate, fluorescein, 2,2′-Azobis(2-amidinopropane) dihydrochloride (AAPH), (±)-6-hydroxy-2,5,7,8-tetramethylchromane-2-carboxylic acid (trolox), gallic acid, methanol (HPLC grade), acetonitrile (LC-MS grade), phosphoric acid 85%, Luperox^®^ TBH70X tert-butyl hydroperoxide solution (TBH), and 2′,7′-Dichlorodihydrofluorescein diacetate (DCFH-DA) were purchased from Sigma-Aldrich (Milan, Italy). CellTiter 96^®^ Aqueous One Solution Assay (MTS) was purchased from Promega (Milan, Italy). The water used in all experiments was Millipore Milli-Q.

### 4.2. Preparation of Cryphaea heteromalla Water Extracts 

*Cryphaea heteromalla* fresh plants, collected on January 2017 in the Trapani area (Sicily, Italy), were repeatedly washed with tap water and lastly three times with distilled water. Next, plants were air-dried for 10 days up to the achievement of a constant weight. Then, the dried plants were crushed in a mortar with liquid nitrogen to obtain a fine powder. Equal amounts of the powder (0.3 g) were mixed with 7.5 mL of three different solvents: pure water (WT), methanol:water (80:20 *v*/*v*) (MET), and ethanol:water (80:20 *v*/*v*) (ETH). Samples were incubated, under magnetic stirring, at 20 °C in the dark, then sonicated for 20 min at room temperature, into an ultrasonic bath at 59 kHz and 198 W. The last step allows a lower degree of antioxidant compound degradation with respect to other techniques [64]. The first extraction was performed up to 4 days to allow for the attainment of the plateau value, indicated by the Folin–Ciocalteau test data performed every 24 h. Further experiments were done using a 3-day extraction time. The extracts were centrifuged at 5000 rpm for 25 min at 4 °C. The supernatants were lyophilized and the obtained powder was stored at –20 °C.

### 4.3. Determination of Total Biophenol Content

The Folin–Ciocalteau assay was performed in agreement with Hrncirik and Frische [65] with a few minor modifications. In brief, 0.2 mL of 1:100 WT, MET, or ETH diluted extracts, were added to 4.8 mL of water, followed by the addition of 0.5 mL Folin–Ciocalteau reagent. After 3 min, 1 mL sodium carbonate solution (20%, *w*/*v*) was added to the reaction mixture, and finally mixed and diluted with water to 10 mL total volume. The absorbance of the mixtures was measured after 2 hours against a blank sample on a Shimadzu Spectrophotometer at a wavelength of 765 nm. A gallic acid calibration curve, linear between 62.5 ÷ 250 mg/mL, was used as a reference. The total biophenol content was expressed as mg of gallic acid equivalents per gram of dried sample.

### 4.4. Oxygen Radical Absorbance Capacity (ORAC) Assay

The method reported by Cao and Prior [66] and Ninfali et al. [67], slightly modified, was applied. The reaction mixture was prepared in the microplate as follows: 160 μL of Fluorescein 0.04 μM in Na–K phosphate buffer 0.075 M (pH 7.0); 20 μL of appropriately diluted extract in WT, MET, or ETH, obtained after 1, 2, and 3 days, or 20 μL of 100 μM Trolox, used as a standard reference. Each mixture was kept for 10 min at 37 °C in the dark, and the reaction was started with the addition of 20 μL of AAPH 40 mM. The fluorescence decay was measured at 37 °C every 5 min at λ = 485 nm excitation and λ = 538 nm emission, using a Thermo Scientific Fluoroskan Ascent F2 Microplate. The ORAC value refers to the net area under the curve of Fluorescein decay in the presence of sample extracts or Trolox, subtracted from the blank area. The activity of the sample was expressed as μmol of Trolox Equivalents (TE)/g of *C. heteromalla* powder, with the following equation:

(1) $$ORAC\;value\;\left({\mu \mathrm{mol}\;\mathrm{TEg}}^{-1}\right)=k\cdot a\cdot h\;\left[\frac{\left(S_{sample}-S_{blank}\right)}{\left(S_{Trolox}-S_{blank}\right)}\right]$$

where *k* is the total dilution of the different extracts; *a* is the ratio between the volume (liters) of the extract and the mass (grams) of dried plants; *h* is the final concentration of Trolox expressed as μmol/L, and S is the area under the curve of Fluorescein in the presence of sample, Trolox, or extraction buffer. 

### 4.5. HPLC-ESI-TOF-MS Chemical Analysis 

Solution at the concentration of 10 g/L was first prepared by dissolving the appropriate amount of extract in water. HPLC analyses were performed with an RRLC 1200 series (Agilent Technologies, Palo Alto, CA, USA), equipped with a vacuum degasser, autosampler, a binary pump, and a DAD detector. The used analytical column was a 150 mm × 4.6 mm i.d., 1.8 µm Zorbax Eclipse Plus C18 (Agilent Technologies, Palo Alto, CA, USA). The used mobile phase was water with 0.5% *v*/*v* acetic acid as the A eluent and acetonitrile as the B eluent. The flow rate was 0.5 mL min^−1^. The total run time was 35 min using the following linear gradient: 0 min, 5% B; 30 min 75% B; 35 min 5% B. The initial condition was held for 5 min. The injection volume was 10 µL and the separation of the compounds was carried out at room temperature. The separated compounds were monitored with a mass spectrometry detector. 

The HPLC system was coupled to a TOF mass spectrometer (Bruker Daltonik, Bremen, Germany) equipped with an orthogonal electrospray (ESI) interface (model G1607 from Agilent Technologies, Palo Alto, CA, USA) operating in negative ionization mode. At this stage, the effluent from the HPLC column was reduced using a “T” type splitter before being introduced into the mass spectrometer (split ratio 1:3). In this sense, the flow arriving to the ESI-TOF-MS detector was 125 µL min^−1^.

The detection was carried out considering a mass range of 50–1000 *m*/*z*. The optimum values of the source parameters were: 4 kV capillary voltage; 210 °C drying gas temperature; drying gas flow, 9 L min^−1^, and nebulizing gas pressure, 2.3 bar. The values of transfer parameters were: capillary exit, 120 V; skimmer 1, 40 V; hexapole 1, 23 V, RF hexapole, 80 Vpp, and skimmer 2, 20 V. The instrument was calibrated externally with a 74900-00-05 Cole Palmer syringe pump (Vernon Hills, IL, USA) that was directly connected to the interface, containing 10 mM sodium acetate cluster solution. The calibration solution was prepared as follows: 10 μL of 1 M sodium hydroxide were mixed with 990 μL of 0.1% *v*/*v* formic acid in water:isopropanol (1:1, *v*/*v*). The mixture was injected at the beginning of each run and all the spectra were calibrated prior to compound identification. To compensate for temperature drift inside the instrument, this external calibration provided accurate mass values with an error lower than 20 ppm.

The accurate mass data of the molecular ions were processed by Data Analysis 4.0 software (Bruker Daltonics, Bremen, Germany), which provided a list of possible elemental formulas by using the Generate Molecular Formula Editor. The Generate Molecular Formula Editor uses a CHNO algorithm, which provides standard functionalities, such as the minimum/maximum elemental range, electron configuration, and ring-plus double bond equivalents, as well as a sophisticated comparison of the theoretical with the measured isotope pattern (mSigma value, see Table 3) for increased confidence in the suggested molecular formula. 

### 4.6. Cell Culture

Mouse embryonic fibroblast NIH-3T3 cells were cultured in Dulbecco’s Modified Eagle Medium (DMEM)-high glucose, supplemented with 100 U/mL penicillin, 100 µg/mL streptomycin and 10% calf bovine serum at 37 °C and 5% CO_2_ humidified atmosphere.

### 4.7. Cell Viability Assay

Cell viability was evaluated by the MTS assay using the CellTiter 96 Aqueous One Solution Cell Proliferation Assay Kit (Promega). NIH-3T3 cells (10^4^ cells/well in 100 µL growth medium) were seeded in a 96-well plate and after 24 h were treated or not (Control) with WT extract at concentrations of 0.25, 0.5, or 1 µg/mL for 24 h. MTS assay was performed according to the manufacturer’s instructions. In brief, after the cell treatments, 20 µL of reagent solution were added into each well. After 4-h incubation at 37 °C in 5% CO_2_, the absorbance was read at 490 nm in a Multiplate reader iMark (BioRad, Hercules, CA, USA). Viability was quantified as percentage relative to untreated cells (Control). 

### 4.8. Protection Analysis against Oxidative Stress

Protective effect of WT extract against oxidative stress was evaluated by the MTS assay. 10^4^ NIH-3T3 cells/well were seeded in a 96-well plate and after 24 h were treated overnight with tert-butyl hydroperoxide (TBH) at 250 µM f.c., or with 0.25 or 0.5 µg/mL of WT extract with or without TBH (250 µM). Untreated cells were used as Control. The MTS assay was then performed as reported above. A microscope (Leica DM IL Inverted Microscope, Wetzlar, Germany) with a camera was used to analyze the morphology of the cells after treatment.

### 4.9. Evaluation of the Cell ROS Content

Intracellular ROS levels were evaluated by DCFH-DA assay accordingly to Wang and Josef [68] with few modifications. In brief, NIH-3T3 cells were seeded in a 96-well black plate at a density of 10^4^ cells/well in 100 µL of phenol red-free culture medium for 24 h. Cells were untreated (Control) or treated overnight with 0.5 µg/mL of WT extract. After the removal of the culture medium, two gentle washes with PBS were done. DCFH-DA was added into each well at 100 µM f.c. and cells were incubated for 1 h at 37 °C. Cells were washed three times with PBS and then treated for 1 h with 500 μM TBH to induce oxidative stress. The latter two steps were performed in phenol red- and serum-free medium. The fluorescence resulting from the production of intracellular ROS was measured in the Fluoroskan Ascent FL Thermo Scientific microplate reader (excitation and emission wavelengths at 485 and 528 nm, respectively). Results were expressed as intracellular ROS levels by measuring the DCF fluorescence intensity. All treatments were performed in the dark.

### 4.10. ROS Detection by Fluorescence Microscopy

Fluorescence microscopy in live cells was performed seeding 2.2 × 10^4^ NIH-3T3 cells/well in a Nunc Lab-Tek II chamber slide in 400 µL of phenol red free culture medium for 24 h. Cells were then untreated (Control) or treated overnight with WT extract at the concentration of 0.5 µg/mL. At the end of the treatment, cells were gently rinsed twice with PBS and incubated for 1 h in phenol red- and serum-free medium with DCFH-DA 100 µM f.c.. Cells were rinsed three times with PBS and treated for 1 h with TBH 500 µM f.c. in phenol red- and serum-free medium. After removal of the medium, cells were rinsed three times with PBS and incubated at room temperature for 10 min with Hoechst 33342 fluorescent DNA-binding dye at 0.01 mg/mL. After rinsing three times with PBS, the chambers were removed and the slide sealed for the microscopic inspection. The nuclei and intracellular ROS levels were detected using a DAPI filter (blue emission) for Hoechst and an FITC filter (green emission) for DCF, respectively, on a Nikon Eclipse 80i microscope equipped for epifluorescence and recorded by a digital camera system.

### 4.11. Statistical Analysis 

All values obtained were reported as the mean of, at least, three independent experiments ± standard deviation (SD). Results were compared using a one-way analysis of variance with pairwise comparisons among treatments made using the Tukey’s HSD test. The analyses were performed using the SigmaPlot 10.0 statistical program (Systat Software Inc., San Jose, CA, USA)

## 5. Conclusions

Numerous studies have shown that plants are a rich source of antioxidants, and different types of solvents from the most polar, e.g., water, to less polar, such as chloroform and hexane, are used to extract them. In our study, water, an eco-friendly and inexpensive solvent, is used to obtain a crude extract with optimum antioxidant activity from the *Cryphaea heteromalla* moss species, which, to the best of our knowledge, was never studied before as a source of bioactive compounds. Further studies will be devoted to the investigation of the nonpolar fraction of this plant.

Bryophyte chemical components can be used in pharmaceutical laboratories as active ingredients for the curing of cancer, and hepatic, cardiovascular, and skin diseases, and other pathologies. The food and cosmetic industries can also discover a powerful source of active molecules in the bryophyte world, taking into account that only a very limited fraction of existing species have been studied and characterized. 

Thus, a positive correlation between folkloristic use and scientific evaluation could generate an alternative source of novel medicinal compounds, which might overcome the expensive research of synthetic drugs and their undesirable side effects. 

## Figures and Tables

**Table 1:** Effects of the solvent composition and extraction times (days) on the total biophenol concentration obtained by *Cryphaea heteromalla*. Concentrations are expressed as mg/g of dry weight.

Extraction Time	WT	MET	ETH
1	2.36 ± 0.04	0.87 ± 0.04	0.66 ± 0.04
2	2.45 ± 0.04	1.00 ± 0.01	0.72 ± 0.03
3	3.34 ± 0.04	1.28 ± 0.02	1.22 ± 0.07
4	3.36 ± 0.07	1.26 ± 0.03	1.18 ± 0.04

**Table 2:** ORAC values obtained as a function of the extraction time (days) and solvent composition. ORAC results are expressed as μmol Trolox equivalent/g dried sample.

Extraction Time	WT	MET	ETH
1	47.33 ± 0.3	21.25 ± 0.5	22.03 ± 0.5
2	50.51 ± 1.0	23.42 ± 0.3	24.32 ± 0.5
3	52.53 ± 1.0	25.36 ± 1.1	26.67 ± 0.5

**Table 3:** Proposed compounds tentatively identified in *Cryphaea heteromalla* WT extract by HPLC-ESI-TOF-MS. The numbers correspond to peaks illustrated in Figure 3A. Compounds numbered as 1, 3, and 12 were not identified.

Peak No.	RT (min)	*m*/*z* Experimental	Molecular Formula (M–H)	*m*/*z* Calculated	Error (ppm)	mSigma	Proposed Compound
**1**	2.64	201.0261	C_4_H_9_O _9_	201.0252	–4.4	149.5	Not identified
**2**	3.9	385.1359	C_14_H_25_O_12_	385.1351	–1.9	17.9	Sugar derivative
**3**	6.22	251.0814	C_9_H_15_O_8_	251.0772	–16.5	4.4	Not identified
**4**	8.46	447.1226	C_18_H_23_O_13_	447.1144	–18.4	10.4	Di-hydroxybenzoic acid hexoside–pentoside
**5**	10.03	473.1386	C_20_H_25_O_13_	473.1301	–18.1	5	Caffeic acid hexoside-pentoside or isomer
**6**	10.84	473.1406	C_20_H_25_O_13_	473.1301	18.3	7.5	Caffeic acid hexoside pentoside or isomer
**7**	19.82	327.2227	C_18_H_31_O_5_	327.2177	–15.3	13.9	Dihydro-*p*-coumaric acid hexoside
**8**	21.02	329.2380	C_18_H_33_O_5_	329.2333	–14.3	5.6	Hydrated-dihydro-*p*-coumaric acid hexoside
**9**	23.01	239.1687	C_14_H_23_O_3_	239.1653	–14.5	10.6	Hydrated-3-oxo-2-(pent-2-enyl)-cyclopentane-1-butanoic acid (hydrated OPC-4)
**10**	24.7	237.1523	C_14_H_21_O_3_	237.1496	–11.5	10	3-Oxo-2-(pent-2-enyl)-cyclopentane-1-butanoic acid (OPC-4)
**11**	25.4	263.1684	C_16_H_23_O_3_	263.1653	–11.9	22	Dinor-oxo-phytodienoic acid
**12**	27.34	315.2569	C_18_H_35_O_4_	315.2541	–8.9	14.1	Not identified
**13**	28.9	265.1836	C_16_H_25_O_3_	265.1809	–10.2	4.9	3-Oxo-2-(2-(Z)-pentenyl) cyclopentane-1-hexanoic acid OPC 6
**14**	29.63	291.1985	C_18_H_27_O_3_	291.1966	–6.8	14.6	*cis*-12-Oxo-phytodienoic acid
